# Retinal Vascularization Abnormalities Studied by Optical Coherence Tomography Angiography (OCTA) in Type 2 Diabetic Patients with Moderate Diabetic Retinopathy

**DOI:** 10.3390/diagnostics12020379

**Published:** 2022-02-01

**Authors:** Guisela Fernández-Espinosa, Ana Boned-Murillo, Elvira Orduna-Hospital, María Dolores Díaz-Barreda, Ana Sánchez-Cano, Sofía Bielsa-Alonso, Javier Acha, Isabel Pinilla

**Affiliations:** 1 Aragon Institute for Health Research (IIS Aragon), 50009 Zaragoza, Spain; 721952@unizar.es (G.F.-E.); abonedm@salud.aragon.es (A.B.-M.); mddiaz@salud.aragon.es (M.D.D.-B.); anaisa@unizar.es (A.S.-C.); j.acha.perez@gmail.com (J.A.); 2 Department of Pharmacology and Physiology, University of Zaragoza, 50009 Zaragoza, Spain; 3 Department of Ophthalmology, Lozano Blesa University Hospital, 50009 Zaragoza, Spain; 4 Department of Applied Physics, University of Zaragoza, 50009 Zaragoza, Spain; 774295@unizar.es; 5 Department of Endocrinology, Miguel Servet University Hospital, 50009 Zaragoza, Spain

**Keywords:** diabetes mellitus, diabetic retinopathy, foveal avascular zone, retinal capillary plexuses, choriocapillaris, swept-source optical coherence tomography angiography, OCTA

## Abstract

Diabetic retinopathy (DR) is the most severe and frequent retinal vascular disease that causes significant visual loss on a global scale. The purpose of our study was to evaluate retinal vascularization in the superficial capillary plexus (SCP), the deep capillary plexus (DCP) and the choriocapillaris (CC) and changes in the foveal avascular zone (FAZ) by optical tomography angiography (OCTA) in patients with type 2 diabetes mellitus (DM2) with moderate DR but without diabetic macular oedema (DME). Fifty-four eyes of DM2 with moderate DR (level 43 in the ETDRS scale) and without DME and 73 age-matched healthy eyes were evaluated using OCTA with swept-source (SS)-OCT to measure microvascularization changes in SCP, DCP, CC and the FAZ. The mean ages were 64.06 ± 11.98 and 60.79 ± 8.62 years in the DM2 and control groups, respectively. Visual acuity (VA) was lower in the DM2 patients (*p* = 0.001), OCTA showed changes in the SCP with a significant diminution in the vascular density and the FAZ area was significantly higher compared to healthy controls, with *p* < 0.001 at the SCP level. The most prevalent anatomical alterations were peripheral disruption in the SCP (83.3%), microaneurysms (MA) in the SCP and in the DCP (79.6% and 79.6%, respectively) and flow changes in the DCP (81.5%). A significant positive correlation was observed between the DM2 duration and the FAZ area in the SCP (0.304 with *p* = 0.025). A significant negative correlation was also found between age and CC central perfusion (*p* < 0.001). In summary, a decrease in the vascular density in DM2 patients with moderate DR without DME was observed, especially at the retinal SPC level. Furthermore, it was found that the FAZ was increased in the DM2 group in both retinal plexuses and was greater in the SCP group.

## 1. Introduction

Diabetic retinopathy (DR) is one of the main causes of blindness worldwide [1]. It is estimated that the prevalence of diabetes mellitus (DM) will increase, reaching 629 million affected people by 2045 [2]. DR is the most severe and frequent ophthalmic complication, related to diabetes macular oedema (DME) and proliferative DR [3].

Currently, the diagnosis and control of DR in daily practice is mainly based on the assessment of visual acuity (VA), the evaluation of the eye fundus, fluorescein angiography (FA) and macular morphology and thickness using optical coherence tomography (OCT) to look for any DR signs. However, despite not finding anatomical retinal changes, visual function could be impaired. New technological advances have improved the OCT resolution for in vivo clinical retinal imaging, especially with the swept-source OCT (SS-OCT) with a resolution of 1050 nm and its unique capability of high performance imaging with high sensitivity and speed relative to spectral-domain OCT (820 nm) [4,5]. SS-OCT allows better evaluation of vascular changes previously unidentified, using OCT angiography (OCTA) to assess retinal and choriocapillaris (CC) microvascularization [6].

OCTA is based on the evaluation of blood flow imaging. The movement of red blood cells generates differences between consecutive B-scans. This provides the opportunity to visualize movement both in the great vessels and in the microvascularization. Thus, it allows the visualization of the superficial (SCP), the intermediate (ICP) and the deep (DCP) capillary plexuses, the radial peripapillary capillary network, the CC and, partially, the choroidal great vessels [6,7].

OCTA can detect multiple abnormalities present in DR, such as capillary nonperfusion areas, microaneurysms (MA) and foveal avascular zone (FAZ) abnormalities in size, morphology or neovascularization (NV). Vascular density (VD), perfusion and FAZ changes are the most frequently investigated OCTA quantitative parameters as predictors of DR since they are correlated with the severity of DR and VA and are useful as treatment markers [8]. OCTA allows the detection of early microvascular changes in DM, even before they are clinically evident in the fundoscopy exam [9,10,11].

The aim of this study was to evaluate the structural changes measured by OCTA in patients with type 2 DM (DM2) and moderate nonproliferative diabetic retinopathy (NPDR) without DME, assess the SCP, DCP and CC, and compare the FAZ values with an age-matched healthy group. To our knowledge, this is the first study including a single level of DR patients DM2 with moderate NPDR since, in previous studies, the diabetic group tended to aggregate different levels of DR [12,13,14,15,16].

## 2. Materials and Methods

### 2.1. Study Design

A prospective single-centre cross-sectional study performed at Ophthalmology Department of the Lozano Blesa University Hospital of Zaragoza was conducted from February 2020 to December 2020, including a total of 127 eyes divided into two groups. Group 1 included 54 eyes of 54 DM2 white patients with moderate DR according to the ETDRS classification (level 43 on the ETDRS retinopathy severity scale) [17] and without DME or other ophthalmological pathology that could compromise best corrected visual acuity (BCVA); Group 2 included 73 eyes of 73 healthy white subjects with no previous history of ocular pathologies, systemic diseases affecting the eye or diabetes family history. The present study complied with the principles of Helsinki and was accepted by the Clinical Research Ethics Committee of Aragon (CEICA PI19/252), and all participants signed informed consent forms.

Exclusion criteria for all participants included amblyopia or BCVA less than 20/40 on the Snellen chart, refractive error over 5.50 diopters (D) of spherical equivalent (SE) or 3.00 D of astigmatism, intraocular pressure (IOP) higher than 20 mmHg, history of any pathology affecting central vision (cataract, age-related macular degeneration (AMD), pathologic myopia (PM), macular hole, macular epiretinal membrane (MEM), macular pucker or traumatic conditions, cerebrovascular accidents, cranioencephalic traumatism or ischemia in carotid territory), glaucoma with perimetric involvement or papillary atrophy, or inability to perform good quality OCT and OCTA evaluation (difficulty in layer segmentation, media opacification, or lack of fixation or cooperation).

### 2.2. Study Protocol

All participants underwent a complete ophthalmological evaluation, including BCVA expressed in logarithm of the minimum resolution angle (logMAR) measured with the ETDRS test, IOP measured by Goldmann tonometry and axial length (AL) using an Aladdin KR-1 W Series optical biometry system (Topcon Corporation, Tokyo, Japan) as the mean of 5 measurements and expressed in millimetres. Clarus imaging was performed (Clarus 700^®^, Carl Zeiss Meditec AG, Jena, Germany). In addition to the ophthalmological evaluation, a complete history was performed in which all aspects related to the patient’s disease (DM2) were collected, including current medication, time of diagnosis, blood glycosylated haemoglobin (HbA1c) levels, serum lipid levels, serum glomerular filtration and serum creatinine levels (the values were obtained within less than 6 months of the examination).

**Figure 1:**
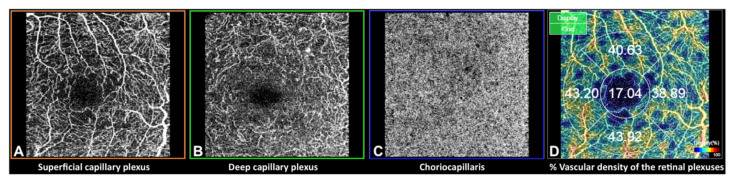
Optical coherence tomography angiography (OCTA) in a 3 × 3 mm macular area of the left eye, measured by deep range imaging (DRI) Triton swept-source (SS)-OCT. (**A**) Superficial capillary plexus, (**B**) deep capillary plexus, (**C**) choriocapillaris and (**D**) an example of vascular density (%) data of the retinal plexuses in 5 areas (abbreviated C: Central, S: Superior, T: Temporal, N: Nasal and I: Inferior).

OCT and OCTA were performed using SS-OCT with deep range imaging (DRI)-Triton SS-OCT (Topcon Corporation, Japan) by the same investigator. To evaluate retinal capillary plexuses and CC VD, the 3 × 3 mm protocol was performed with IMAGEnet 6 Version software 1.22.1.14101^®^ 2014 (Topcon Corporation, Japan). DRI Triton images the SCP, the DCP and the CC and gives its VD values in a grid divided into central (C), superior (S), inferior (I) temporal (T) and nasal (N) quadrants in % pixels occupied by vessels (Figure 1). The FAZ area was measured manually using the measurement tool in both the SCP and DCP. All of the images were analysed by two different readers (IP, ABM) looking for vascular abnormalities. In case of disagreement, images were evaluated by both readers who reached an agreement.

### 2.3. Statistical Analysis

Statistical analysis was performed using the Statistical Package for the Social Sciences software (SPSS version 20, SPSS Inc., IBM Corporation, Armonk, NY, USA). First, a descriptive and frequency analysis of the sample was carried out according to the demographic variables and clinical characteristics. Second, the structural changes were determined by assessing the SCP, DCP and CC, as well as the FAZ values, to compare the results with the healthy group. The normal distribution of the values was studied with the Kolmogorov–Smirnov test, and, subsequently, the Mann–Whitney U test was performed for independent nonparametric samples to assess if there were statistically significant differences between the groups. For the correlation of the anatomical results and the disease control parameters, a bivariate analysis was performed using the Spearman correlation test. In DM patients, OCTA anatomical changes were described as % of the total, considering 0% as the absence of the anatomical alteration and 100% as the maximum presence of it. For all analyses, a value of *p* < 0.05 was considered statistically significant.

## 3. Results

The mean age of the 54 DM2 patients was 64.06 ± 11.98 years (42–86) and 60.79 ± 8.62 years (42–83) for the 73 healthy controls, without a significant age difference (*p* = 0.082). Groups were sex-matched, men were 61.11% (*n* = 33) and 60.30% (*n* = 44) of the DM and control groups, respectively; women were 38.89% (*n* = 21) and 39.70% (*n* = 29) of the DM and control groups, respectively. In the DM2 group, the mean time since diagnosis was 2.50 ± 2.88 years (range 0–11 years), with good glycaemic control (HbA1c = 7.58 ± 1.30%). Glycaemic, lipid and renal function values are presented in Table 1.

There were no differences between groups in their AL (*p* = 0.075), SE (*p* = 0.110) or IOP (*p* = 0.676). BCVA taken with the 100% contrast ETDRS test reached statistical significance (*p* = 0.001), with a lower VA in the DM2 group (0.10 ± 0.12 LogMAR vs. 0.04 ± 0.05 LogMAR in the DM2 group vs. in the healthy group, respectively) (Table 2).

### 3.1. Vascular Density and Microvascular Changes Studied with OCTA

**Figure 2:**
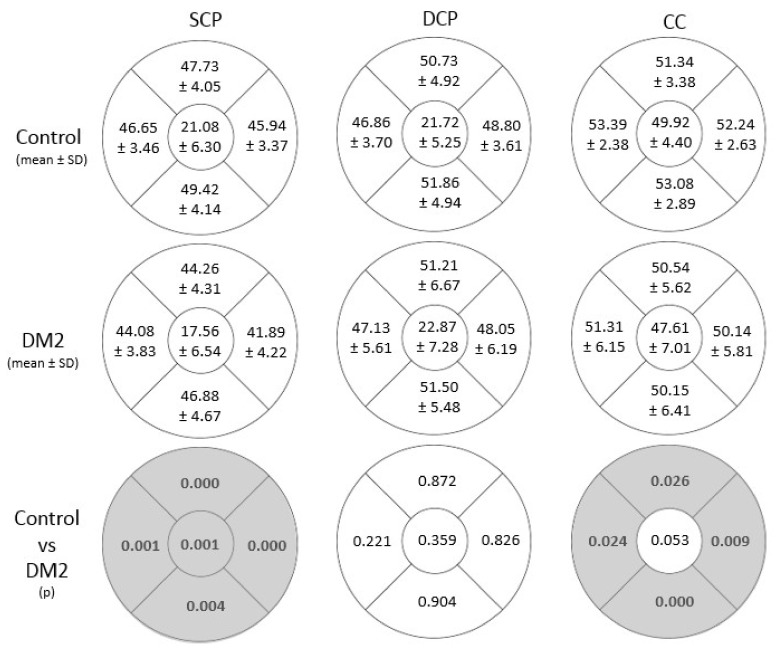
Mean and standard deviation (SD) of vascular density (%) of the superficial (SCP) and deep (DCP) retinal plexuses, the choriocapillaris (CC), measured using DRI Triton SS-OCT in patients with DM2 and in healthy controls and their comparison. The measurements were divided into 5 quadrants (abbreviated C, Central; S, Superior; T, Temporal; N, Nasal; I, Inferior). Differences that reached statistical significance (*p* < 0.05) are shown in grey.

The DM2 group presented a significantly lower VD in the SCP than the control group in all regions (C, S, T, N and I areas). The values are presented in Figure 2 (*p* < 0.05).

Regarding the rest of the VD values analysed, no statistically significant differences were found in the DCP. However, statistically significant differences were found in the CC (Figure 2) in the S (51.34 ± 3.38% in controls vs. 50.55 ± 5.62% in the DM2 group with *p* = 0.026), T (53.39 ± 2.38% in controls vs. 51.31 ± 6.15% in the DM2 group with *p* = 0.024), N (52.24 ± 2.63% in controls vs. 50.14 ± 5.81% in the DM2 group with *p* < 0.009) and I (53.08 ± 2.89% in controls vs. 50.15 ± 6.41% in the DM2 group with *p* < 0.001) areas. The CC C area showed higher values in the control group without reaching statistical significance (*p* = 0.053).

The FAZ area of the SCP was significantly higher in the DM2 group than in the control group (242.37 ± 85.36 μm^2^ in the control group vs. 333.58 ± 161.03 μm^2^ in the DM2 group with *p* < 0.001). We did not find differences in the FAZ area of the DCP (Table 3).

**Figure 3:**
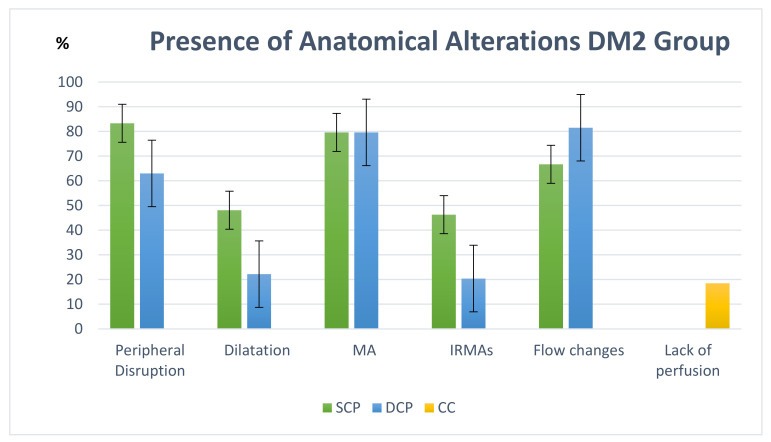
Qualitative analysis of the alterations presented in the type 2 diabetes mellitus (DM2) group in percentage (%). Abbreviations: SCP, superficial capillary plexus; MA, microaneurysms; IRMAs, intraretinal microvascular abnormalities; DCP, deep capillary plexus; CC, choriocapillaris.

**Figure 4:**
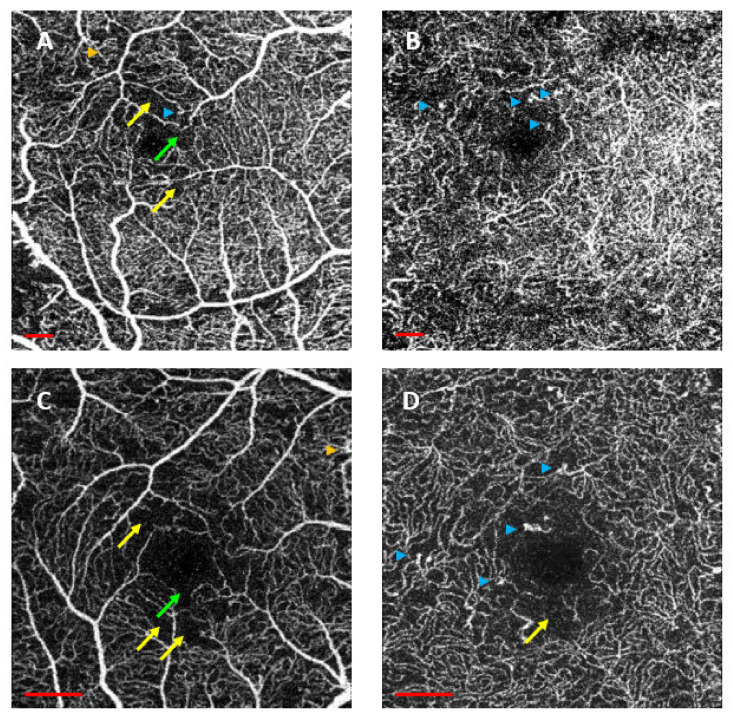
Anatomical changes in the type 2 diabetes mellitus (DM2) group imaged by optical coherence tomography angiography (OCTA). (**A**,**B**) correspond to the superficial capillary plexus (SCP) and deep capillary plexus (DCP) in a 6 × 6 scan and (**C**,**D**) to the SCP and DCP in a 3 × 3 scan protocol. (**A**,**B**) show microaneurysms in both the SCP (**A**) and DCP (**B**). (**C**,**D**) show foveal avascular zone (FAZ) disruption, perfusion loss and intraretinal microvascular abnormalities (IRMAs) in both the SCP (**C**) and DCP (**D**). Microaneurysms are shown as blue arrowheads, FAZ disruption as green arrows, nonperfusion areas as yellow arrows and IRMA as orange arrowhead. Scale bar (in red) represents 500 µm.

We studied anatomical alterations, including peripheral disruption of the FAZ, linear vascular dilatations, MA, intraretinal microvascular abnormalities (IRMAs), flow changes and lack of CC perfusion. Changes were present in both plexuses. FAZ disruption and IRMAs were more frequently detected in the SCP. Perfusion changes and MA were similar in both plexuses. The most prevalent anatomical alterations in the DM2 group were peripheral disruption in the SCP (83.3%), MA in the SCP and in the DCP (79.6% and 79.6%, respectively) and flow changes in the DCP (81.5%) (Figure 3). Figure 4 shows an example of the anatomical changes in the DM2 group. The percentages of the presence or absence of these alterations in the DM2 group are shown in Figure 3.

### 3.2. Anatomo-Functional Correlation Study

We used the Spearman test to correlate the structural and functional data obtained by DRI Triton SS-OCT and the qualitative analysis of the alterations present in DM2.

**Figure 5:**
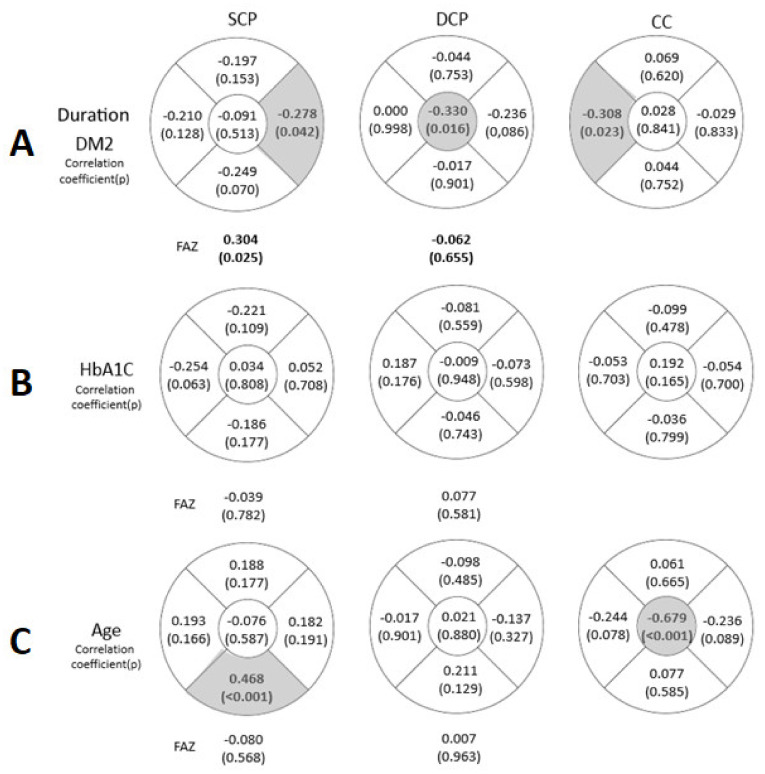
Correlation between type 2 diabetes mellitus (DM2) duration (**A**), glycosylated haemoglobin (HbA1c) (%) (**B**) and age (**C**) in patients with DM2 in the 5 areas and in the FAZ of the SCP, DCP and CC. The values that reached statistical significance (*p* < 0.05) are shown in grey.

Regarding the DM2 duration, there was a significant negative correlation (*p* < 0.05) in the N area of the SCP (−0.278, *p* = 0.042), in the C area of the DCP (−0.330, *p* = 0.016) and in the T area of the CC (−0.308, *p* = 0.023). A positive correlation was obtained between the DM2 duration and the FAZ area in the SCP (0.304 with *p* = 0.025) (Figure 5).

Regarding patient age, there was a negative correlation (*p* < 0.001) with the C area of the CC.

Table 4 shows the correlation between anatomical alterations in DM2 and visual function, disease duration and metabolic control. We found a significant negative correlation between high-density lipoprotein (HDL) levels and IRMAs (weak correlation: −0.308, *p* = 0.024) and flow changes (moderate correlation: −0.415, *p* = 0.002) in the SCP.

Table 5 shows the correlation between the anatomical changes at the retinal plexuses, at the CC, at the VD in the 5 sectors (C, S, T, N and I) and in the FAZ area in the SCP and DCP.

Significant negative correlations were observed in the T and N sectors of the DCP and the presence of flow changes in this plexus (−0.290; *p* = 0.034 and −0.348; *p* = 0.010, respectively). In addition, negative correlations were found between the C and N sectors of the CC and the presence of a lack of perfusion (−0.349; *p* = 0.010 and −0.309; *p* = 0.023, respectively) (Table 5).

## 4. Discussion

There are different studies evaluating the use of OCTA to analyse different findings in DR, with special emphasis on the FAZ size, morphology and capillary perfusion [18,19,20]. Macular microvascularization has been related to proliferative retinopathy and DME and is associated with nonperfusion areas and increased leakage [21]. We studied a group of patients with only moderate DR (level 43 on the ETDRS scale) to evaluate changes in their vessels. We excluded patients with DME to avoid any potentially confusing effects in the OCTA metrics [22].

At the beginning of the study, we verified that the groups were similar in terms of variables that could alter the results, such as sex and age or AL, and with IOP in the normal range. These values can modify the data obtained by OCT and OCTA, not only in the choroidal thickness and in the CC VD [23] but also in the macular perfusion with a progressive VD diminution and an increase in the FAZ area [24,25,26]. The FAZ area has also been related to other factors, such as central retinal thickness, sex, SE, AL and choroidal thickness [27]. OCTA studies provide different findings not only in normal eyes but also in DM patients. This can be related to different methodological techniques, measurements or devices [28,29] and the stage of DM disease or its duration. Age has also been described as a confounding factor in OCTA and DM patients. Ghassemi et al. [28] found a negative correlation between age and parafoveal VD in the SCP in DM. In our study, we found a negative correlation between age and CC perfusion, indicating a diminution of CC flow with age. We had a higher proportion of women in the non-DM group, but we do not expect this difference to have affected our results.

All patients had diabetic control close to HbA1c 7% and preserved renal function; impaired renal function could affect retinal thickness and cause oedema.

Regarding BCVA, in our study, significant differences (*p* = 0.001) were obtained between groups, with lower VA in the DM2 group (0.10 ± 0.12 LogMAR vs. 0.04 ± 0.05 LogMAR in the DM2 group vs. the healthy one). Zhu et al. [30] presented similar results of BCVA (0.10 ± 0.19 LogMAR) in DM2, but they found no differences from the control group. In another follow-up study, the initial BCVA in DM1 patients was −0.03 ± 0.16 LogMAR, but it had worsened to 0.03 ± 0.20 LogMAR 10 years later [31]. The BCVA results were better in Orduna et al.’s paper [32] studying a younger DM1 group without DR (−0.13 ± 0.11 LogMAR, *p* = 0.910) with the same 100% contrast ETDRS test; the good BCVA values were probably related to the mean age of the DM1 group (41.52 ± 13.05 years). BCVA decreases with disease duration, probably due to the retinal neurodegeneration theory, which has been related to less perfusion and higher metabolic demands of the inner retina that make it more vulnerable to diabetes-induced metabolic stress [33] and worse visual function; functional impairments have been especially described for contrast sensitivity [34], colour perception [1] and dark adaptation [35].

In our study, the OCTA results showed a decrease in VD in all areas of the SCP of the DM2 group (C, S, T, N and I) and most areas of the CC (S, T, N and I) with significant differences with respect to the healthy controls (Figure 2), which may be related to the BCVA decrease observed in these patients. However, these differences were not present in the DCP. Different studies found a reduction in VD in the diabetic group versus the healthy group, not only in the SCP but also in the DCP and CC. Forte et al. [36] and Alam et al. [37] in 2020 found lower VD in the DM2 group with a decrease in VD with DR progression. Ghassemi et al. also described lower VD in both retinal plexuses in DM patients than in normal eyes [28]. This reduction progressed with the worsening of DR. Other authors, such as Nesper et al. [38] or Dimitrova et al. [39], described a higher impairment in the DCP with the progression of the disease.

However, Ong et al. [40] described the utility of VD evaluation, the FAZ and the vessel length density at the SCP to distinguish healthy subjects and the different stages of nonproliferative DR (NPDR). They suggested that SCP changes are more reliable due to the lower noise and artefacts in OCTA acquisition. They found less variability in the vessel length skeleton at the DCP in moderate to severe NPDR. Our study was based only on DM2 patients with a moderate NPDR, without comparisons with other stages and not pooling with other stages of DR. Other authors, such as Durbin et al. [41], also found that VD in the SCP had the highest area under the receiver operating characteristic (ROC) curve, indicating that it is the best method to distinguish between diabetic and nondiabetic eyes. In their study, they pooled moderate and severe NPDR together compared to early stage and mild DR pooled together.

DM has manifestations at the choroidal level, with findings not only in anatomical studies but also in indocyanine green angiography and OCT [42,43,44,45,46]. It is straightforward to find manifestations in the CC. Understanding choroidal changes can help to predict disease progression [47]. In our study, we also found significant differences in VD in parafoveal sectors (S, T, N and I), excluding the central sector. Our findings are similar to those authors describing a pathological choroid in DM patients related to their renal function [48]. Zhang et al. [49] described a higher choroidal thickness with lower CC flow using OCTA.

Regarding the FAZ, we observed a significantly greater area at the SCP level in the DM2 group; however, no significant differences were found in DCP. Despite not reaching statistical significance in the DCP, we observed that the FAZ area increased in both plexuses in the DM2 group compared to the control group. Our results are similar to other authors, such as De Carlo [50] et al. and Dimitrova et al. [39], who demonstrated an increase in the FAZ size of the SCP in DM patients without DR associated with a decrease in VD in both plexuses. Takase et al. [51] and Di et al. [52] found that patients with more severe retinal damage had a larger FAZ and that the FAZ can be used to predict DR progression and DME and VA impairment. Otherwise, clinical examinations and glycaemic control still have the primary role in detecting preclinical changes in patients with diabetes.

Other authors, such as Lupidi et al. [18], have studied anatomical alterations in DM patients in different plexuses. They studied DM1 and DM2 patients with nonproliferative RD and no DME, pooling different stages. Their results were similar to ours. They described abnormalities in both SCP and DCP. They found a higher number of linear vascular dilatations and a smaller number of MAs (in our series, MA was described in both plexuses in 80% of the patients, but 55% and 64% were described in SCP and CP, respectively). Peripheral disruption and flow changes were almost equal in both studies. Couturier et al. [53] found a lack of perfusion in the SCP. In the DCP, they found a lack of perfusion in some eyes and MA, although these were better detected by fluorescein angiography. Hwang et al. [54] also found a lack of capillary perfusion in the SCP and in the DCP, in addition to vascular dilatations in the DCP.

Although we were not able to find a VD diminution in the DCP in the DM2 group (Figure 2), we found a negative correlation, not only in the N area of the SCP and in the T area of the CC but also in the C area of the DCP with the DM2 duration (Figure 5). Xie et al. [55] described a lower VD associated with age and higher levels of HbA1c. Our DM2 patients had a short disease duration (2.50 ± 2.88 years, range 0–11 years), which could explain the smaller changes in the VD at the DCP or they had an early diagnosis, before the signs became more severe. These results suggest that VD diminished with the progression of DM2, its duration and with the aging of the patient, as described by Lavia et al. [56] and Ciloglu et al. [57]. Most likely, this loss in the C area of the DCP will be reflected over time by an increase in the FAZ area in the DCP and a potential decrease in the BCVA. Other studies did not find any correlation between the VD or FAZ area and HbA1c or the duration of the disease [41].

We also found a significant negative correlation between HDL levels and anatomical alterations of the SCP (IRMAs and flow changes), suggesting a vascular implication of dyslipidaemia. Additionally, a significant positive correlation was found between AL and flow changes in the SCP, with greater flow changes with higher AL values. Tang et al. [58] described a lower VD associated with a shorter AL as well as a more severe and worse VA.

The use of artificial intelligence (AI) or deep learning would help the evaluation of OCTA images in different DR levels. Xiang et al. and Nazir et al. have demonstrated the ability of the technique to distinguish DR severity. In the near future, OCTA will help to conduct the DR progression assessment using AI [59,60,61].

## 5. Conclusions

In conclusion, retinal vascularization is affected in DM2 patients with moderate DR without DME. There was a diminution in VD and an increase in the FAZ area, especially in the SCP. DM2 patients with moderate DR show anatomical alterations in both plexuses. OCTA provides powerful information about retinal vascularization and the FAZ area, depending on the DR level.

## Figures and Tables

**Table 1:** Mean and standard deviation (SD) of the diabetic disease and systemic values (lipid levels and renal function) of the type 2 diabetic mellitus (DM2) patients. Abbreviations: DM2, diabetes mellitus type 2; HbA1c (%), glycosylated haemoglobin; HDL, high-density lipoprotein; LDL, low-density lipoprotein; TG, triglycerides; GF, glomerular filtration.

DM2 Group (*n* = 54)	Mean	SD
Disease duration (years)	2.50	2.88
HbA1c (%)	7.58	1.30
Total cholesterol total (mg/dL)	148.04	33.18
HDL cholesterol HDL (mg/dL)	47.83	15.21
LDL cholesterol LDL (mg/dL)	71.47	23.10
TG (mg/dL)	122.24	51.71
GF (CKD-EPI) (mL/min/1.73)	73.57	20.52
Creatinine (mg/dL)	1.05	0.50

**Table 2:** Mean, standard deviation (SD) and statistical significance (*p* value) of the best corrected visual acuity (BCVA) in the LogMAR scale, spherical equivalent (SE) in diopters (D), axial length (AL) in mm and intraocular pressure (IOP) in mmHg between the control and type 2 diabetes mellitus (DM2) groups. Differences that reached statistical significance with *p* < 0.05 are shown in bold.

	Control Group (*n* = 73)		DM2 Group (*n* = 54)		*p*
	Mean	SD	Mean	SD	
BCVA (LogMAR)	0.04	0.05	0.10	0.12	**0.001**
SE (D)	0.04	1.58	0.37	1.70	0.110
AL (mm)	24.00	2.80	23.23	0.84	0.075
IOP (mmHg)	15.30	2.90	14.76	2.49	0.676

**Table 3:** Mean and standard deviation (SD) of the foveal avascular zone (FAZ) (μm^2^) of the superficial (SCP) and deep (DCP) retinal plexuses. Differences that reached statistical significance (*p* < 0.05) are shown in bold.

FAZ Area (μm^2^)					
	Control Group		DM2 Group		*p*
	Mean	SD	Mean	SD	
SCP	242.37	85.36	333.59	161.03	**<0.0001**
DCP	278.85	103.25	307.18	141.16	0.301

**Table 4:** Correlation coefficient and *p*-value (in brackets) of the anatomical alterations found in type 2 diabetes mellitus (DM2) with the different studied variables of visual function, disease duration and metabolic control. The values that reached statistical significance (* *p* < 0.05 and ** *p* < 0.01) are shown in bold. Abbreviations: SCP, superficial capillary plexus; DCP, deep capillary plexus; CC, choriocapillaris; MA, microaneurysms; IRMAs, intraretinal microvascular abnormalities; DM2, diabetes mellitus type 2; HbA1c (%), glycosylated haemoglobin; HDL, high-density lipoprotein; LDL, low-density lipoprotein; TG, triglycerides; GF, glomerular filtration; VA, visual acuity; LogMAR, logarithm of the minimum resolution angle; SE, spherical equivalent; D, diopters; AL, axial length; IOP, intraocular pressure.

Anatomical Alterations in NPDR DM2 Patients											
	Peripheral Disruption SCP	Dilatation SCP	MA SCP	IRMAs SCP	Flow Changes SCP	Peripheral Disruption DCP	Dilatation DCP	MA DCP	IRMAs DCP	Flow Changes DCP	Lack of CC Perfusion
Age	−0.082 (0.558)	−0.027 (0.847)	0.081 (0.565)	−0.162 (0.246)	0.080 (0.571)	−0.043 (0.762)	0.286 * (0.038)	−0.122 (0.386)	−0.182 (0.193)	0.096 (0.493)	0.251 (0.070)
Duration DM2	0.094 (0.500)	0.113 (0.417)	0.010 (0.945)	0.130 (0.349)	0.202 (0.143)	−0.092 (0.506)	0.254 (0.064)	−0.021 (0.881)	−0.056 (0.686)	−0.025 (0.858)	−0.075 (0.590)
HbA1C%	−0.008 (0.954)	0.111 (0.425)	−0.064 (0.648)	0.138 (0.318)	−0.040 (0.772)	0.042 (0.764)	−0.186 (0.178)	0.066 (0.633)	−0.052 (0.710)	−0.155 (0.264)	−0.052 (0.708)
Cholesterol	0.059 (0.672)	−0.092 (0.510)	−0.084 (0.545)	−0.236 (0.086)	−0.189 (0.171)	0.022 (0.874)	0.020 (0.886)	0.133 (0.338)	0.084 (0.545)	−0.240 (0.080)	−0.121 (0.384)
HDL	0.067 (0.630)	−0.241 (0.080)	−0.155 (0.263)	−0.308 * (0.024)	−0.415 ** (0.002)	−0.076 (0.583)	−0.176 (0.203)	−0.096 (0.490)	−0.015 (0.916)	−0.078 (0.575)	−0.225 (0.102)
LDL	−0.033 (0.810)	−0.020 (0.885)	−0.072 (0.603)	−0.248 (0.071)	−0.262 (0.055)	0.009 (0.951)	−0.044 (0.750)	0.198 (0.152)	0.046 (0.743)	−0.219 (0.112)	−0.106 (0.447)
TG	−0.217 (0.115)	0.104 (0.456)	0.254 (0.064)	0.181 (0.190)	0.077 (0.580)	0.108 (0.436)	0.159 (0.252)	0.080 (0.567)	0.108 (0.438)	−0.047 (0.733)	0.008 (0.956)
GF	0.198 (0.152)	0.080 (0.563)	0.117 (0.401)	0.200 (0.148)	0.018 (0.895)	−0.037 (0.790)	−0.061 (0.661)	0.238 (0.083)	0.258 (0.060)	−0.275 * (0.044)	−0.328 * (0.015)
Creatine	−0.097 (0.484)	−0.007 (0.959)	−0.123 (0.377)	−0.107 (0.440)	0.098 (0.479)	0.103 (0.457)	0.034 (0.805)	−0.227 (0.098)	−0.205 (0.137)	0.271 * (0.048)	0.305 * (0.025)
VA (LogMAR)	−0.051 (0.716)	−0.111 (0.426)	−0.220 (0.110)	−0.076 (0.587)	−0.065 (0.639)	−0.017 (0.903)	0.009 (0.948)	−0.027 (0.849)	−0.039 (0.779)	−0.094 (0.500)	0.165 (0.233)
SE (D)	−0.053 (0.704)	0.140 (0.314)	−0.033 (0.815)	−0.051 (0.712)	−0.134 (0.333)	−0.200 (0.147)	−0.142 (0.305)	−0.135 (0.331)	0.030 (0.832)	0.214 (0.121)	−0.003 (0.982)
AL (mm)	0.115 (0.408)	−0.180 (0.194)	0.089 (0.524)	−0.054 (0.700)	0.421 ** (0.002)	0.073 (0.602)	−0.014 (0.918)	0.136 (0.328)	0.199 (0.149)	0.144 (0.300)	0.024 (0.861)
IOP (mmHg)	−0.003 (0.982)	0.112 (0.421)	0.039 (0.781)	−0.218 (0.113)	−0.149 (0.282)	−0.090 (0.520)	0.092 (0.506)	−0.172 (0.215)	0.015 (0.915)	−0.186 (0.179)	−0.315 * (0.020)

**Table 5:** Correlation coefficient and *p*-value (in brackets) of the anatomical alterations in type 2 diabetes mellitus (DM2) in the SCP, DCP and CC in the 5 sectors and in the FAZ. The values that reached statistical significance (* *p* < 0.05 and ** *p* < 0.01) are shown in bold. Abbreviations: SCP, superficial capillary plexus; DCP, deep capillary plexus; CC, choriocapillaris; MA, microaneurysms; IRMAs, intraretinal microvascular abnormalities; C, Central; S, Superior; T, Temporal; N, Nasal; I, Inferior; FAZ, foveal avascular zone.

Anatomical Alterations in NPDR DM2 Patients											
	Peripheral Disruption SCP	Dilatation SCP	MA SCP	IRMAs SCP	Flow Changes SCP	Peripheral Disruption DCP	Dilatation DCP	MA DCP	IRMAs DCP	Flow Changes DCP	Lack of CC Perfusion
**SCP**											
C	0.018 (0.900)	0.033 (0.811)	0.199 (0.149)	−0.113 (0.415)	−0.048 (0.731)	0.212 (0.125)	−0.054 (0.697)	0.081 (0.560)	0.136 (0.328)	0.052 (0.709)	0.101 (0.468)
S	0.172 (0.213)	−0.260 (0.057)	−0.155 (0.263)	−0.318 * (0.019)	−0.130 (0.350)	−0.148 (0.287)	0.101 (0.465)	−0.223 (0.105)	−0.103 (0.457)	0.000 (1.000)	−0.217 (0.115)
T	−0.091 (0.513)	0.002 (0.986)	0.117 (0.401)	−0.135 (0.332)	−0.272 * (0.046)	−0.076 (0.584)	0.049 (0.727)	−0.155 (0.263)	−0.043 (0.759)	0.064 (0.644)	0.021 (0.878)
N	0.014 (0.918)	−0.112 (0.421)	0.024 ± 0.865	−0.166 (0.231)	−0.212 (0.124)	−0.074 (0.596)	−0.170 (0.219)	−0.060 (0.664)	−0.056 (0.687)	−0.014 (0.921)	0.185 (0.180)
I	0.081 (0.559)	−0.026 (0.851)	0.007 ± 0.958	−0.139 (0.315)	0.018 (0.899)	−0.042 (0.764)	0.123 (0.376)	−0.040 (0.775)	−0.235 (0.088)	0.113 (0.415)	0.084 (0.545)
FAZ	0.002 (0.991)	0.221 (0.108)	−0.040 (0.775)	0.142 (0.306)	0.058 (0.677)	−0.093 (0.501)	0.003 (0.984)	−0.013 (0.924)	−0.009 (0.949)	0.107 (0.441)	0.037 (0.792)
**DCP**											
C	−0.010 (0.944)	−0.035 (0.806)	0.131 (0.351)	−0.285 * (0.039)	−0.172 (0.218)	0.008 (0.957)	−0.068 (0.630)	0.079 (0.574)	−0.002 (0.991)	−0.195 (0.161)	0.142 (0.311)
S	0.143 (0.301)	−0.284 * (0.037)	−0.168 (0.224)	−0.193 (0.162)	−0.200 (0.146)	−0.204 (0.139)	−0.033 (0.813)	−0.108 (0.438)	0.062 (0.656)	−0.167 (0.228)	−0.026 (0.852)
T	−0.180 (0.192)	0.243 (0.077)	−0.058 (0.679)	0.035 (0.804)	−0.290 * (0.034)	−0.155 (0.263)	0.020 (0.886)	−0.031 (0.824)	−0.019 (0.891)	−0.199 (0.150)	0.141 (0.310)
N	−0.126 (0.364)	−0.065 (0.638)	0.037 (0.791)	−0.127 (0.358)	−0.348 ** (0.010)	−0.207 (0.134)	−0.016 (0.910)	0.041 (0.767)	−0.090 (0.518)	−0.382 ** (0.004)	0.073 (0.598)
I	0.069 (0.622)	0.078 (0.573)	−0.162 (0.241)	−0.074 (0.596)	−0.103 (0.457)	−0.044 (0.750)	0.049 (0.727)	0.000 (1.000)	−0.208 (0.131)	0.156 (0.260)	0.127 (0.360)
FAZ	−0.069 (0.622)	−0.045 (0.746)	−0.205 (0.137)	0.030 (0.831)	0.149 (0.283)	−0.042 (0.764)	−0.069 (0.622)	−0.205 (0.137)	0.046 (0.743)	0.229 (0.095)	0.049 (0.725)
**CC**											
C	0.065 (0.639)	0.102 (0.462)	−0.074 (0.596)	0.018 (0.898)	−0.151 (0.275)	0.197 (0.154)	−0.157 (0.256)	0.058 (0.679)	0.173 (0.212)	0.021 (0.878)	−0.349 ** (0.010)
S	0.118 (0.396)	0.114 (0.411)	−0.090 (0.518)	0.139 (0.315)	0.165 (0.233)	0.140 (0.312)	0.100 (0.472)	−0.010 ± 0.941	−0.068 ± 0.626	0.179 ± 0.195	−0.116 (0.403)
T	−0.086 (0.536)	0.170 (0.219)	0.128 (0.355)	−0.046 (0.739)	−0.377 ** (0.005)	0.278 * (0.042)	−0.252 (0.067)	0.226 ± 0.101	0.077 ± 0.581	−0.145 ± 0.295	−0.177 (0.199)
N	−0.018 (0.900)	−0.100 (0.472)	−0.193 (0.162)	−0.244 (0.075)	−0.237 (0.085)	0.108 (0.436)	−0.300 * (0.027)	0.034 ± 0.808	−0.072 ± 0.603	0.037 ± 0.792	−0.309 * (0.023)
I	0.171 (0.218)	−0.064 (0.645)	−0.075 (0.589)	−0.087 (0.532)	0.197 (0.154)	0.032 (0.818)	−0.089 (0.524)	0.031 ± 0.824	−0.264 ± 0.054	0.162 ± 0.242	−0.080 (0.568)

## Data Availability

The data presented in this study are available within the article.
